# Acoustilytix™: A Web-Based Automated Ultrasonic Vocalization Scoring Platform

**DOI:** 10.3390/brainsci11070864

**Published:** 2021-06-29

**Authors:** Catherine B. Ashley, Ryan D. Snyder, James E. Shepherd, Catalina Cervantes, Nitish Mittal, Sheila Fleming, Jaxon Bailey, Maisie D. Nievera, Sharmin Islam Souleimanova, Bill Nyaoga, Lauren Lichtenfeld, Alicia R. Chen, W. Todd Maddox, Christine L. Duvauchelle

**Affiliations:** 1 Cornerstone Research Group, Miamisburg, OH 45342, USA; ashleycb@crgrp.com (C.B.A.); snyderrd@crgrp.com (R.D.S.); shepherdje@crgrp.com (J.E.S.); 2 Division of Pharmacology and Toxicology, College of Pharmacy, The University of Texas at Austin, 2409 University Avenue, Stop A1915, Austin, TX 78712, USA; catalinacervantes@austin.utexas.edu (C.C.); jaxon.bailey@utexas.edu (J.B.); maisie.nievera@utexas.edu (M.D.N.); sharmin.souleimanova@austin.utexas.edu (S.I.S.); bill.nyaoga@austin.utexas.edu (B.N.); lauren.lichtenfeld@austin.utexas.edu (L.L.); alicia.chen@austin.utexas.edu (A.R.C.); 3 ZS Associates, Empire State Building, 350 Fifth Avenue, Suite 5100, New York, NY 10118, USA; niimits1@gmail.com; 4 Department of Pharmaceutical Sciences, Northeast Ohio Medical University, 4209 State Route 44, RGE, Rootstown, OH 44272, USA; sfleming1@neomed.edu; 5 Cognitive Design and Statistical Consulting, LLC, Austin, TX 78746, USA; wtoddmaddox@gmail.com; 6 Waggoner Center for Alcohol and Addiction Research, The University of Texas at Austin, 2500 Speedway, Stop A4800, Austin, TX 78712, USA

**Keywords:** ultrasonic vocalization, automated scoring, dopamine, addiction, mental health, machine learning, drug discovery, drug development

## Abstract

Ultrasonic vocalizations (USVs) are known to reflect emotional processing, brain neurochemistry, and brain function. Collecting and processing USV data is manual, time-intensive, and costly, creating a significant bottleneck by limiting researchers’ ability to employ fully effective and nuanced experimental designs and serving as a barrier to entry for other researchers. In this report, we provide a snapshot of the current development and testing of Acoustilytix™, a web-based automated USV scoring tool. Acoustilytix implements machine learning methodology in the USV detection and classification process and is recording-environment-agnostic. We summarize the user features identified as desirable by USV researchers and how these were implemented. These include the ability to easily upload USV files, output a list of detected USVs with associated parameters in csv format, and the ability to manually verify or modify an automatically detected call. With no user intervention or tuning, Acoustilytix achieves 93% sensitivity (a measure of how accurately Acoustilytix detects true calls) and 73% precision (a measure of how accurately Acoustilytix avoids false positives) in call detection across four unique recording environments and was superior to the popular DeepSqueak algorithm (sensitivity = 88%; precision = 41%). Future work will include integration and implementation of machine-learning-based call type classification prediction that will recommend a call type to the user for each detected call. Call classification accuracy is currently in the 71–79% accuracy range, which will continue to improve as more USV files are scored by expert scorers, providing more training data for the classification model. We also describe a recently developed feature of Acoustilytix that offers a fast and effective way to train hand-scorers using automated learning principles without the need for an expert hand-scorer to be present and is built upon a foundation of learning science. The key is that trainees are given practice classifying hundreds of calls with immediate corrective feedback based on an expert’s USV classification. We showed that this approach is highly effective with inter-rater reliability (i.e., kappa statistics) between trainees and the expert ranging from 0.30–0.75 (average = 0.55) after only 1000–2000 calls of training. We conclude with a brief discussion of future improvements to the Acoustilytix platform.

## 1. Introduction

Identifying neural mechanisms underlying mental health and addiction disorders is necessary to develop successful interventions, but moving from basic science findings to clinical trials is a lengthy and high-risk process. Although not the sole determinant, emotional processing is directly involved in many mental health disorders and addictions. Animal models allow direct measurement and manipulation of the neural circuitry that drives emotional processing, circuitry that often can only be inferred in human studies [1,2,3,4,5].

Rodent ultrasonic vocalizations (USVs) are known to reflect emotional processing, brain neurochemistry, and brain function-key observations in animal model studies. USVs in the 22–28 kHz and 50–55 kHz frequency range are widely recognized forms of social and emotional expression in rats [6]. USVs in the 22–28 kHz range are provoked by external threats (e.g., danger) or unpleasant internal conditions (e.g., sickness, pain) [6,7,8]. These negative-affect associated 22–28 kHz USVs are initiated through the ascending mesolimbic cholinergic pathway, originating in the laterodorsal tegmental nucleus (LDT) and extending through the midbrain, anterior hypothalamic, preoptic, and septal regions [7,9,10]. Positive-affect associated USVs, commonly referred to as 50–55 kHz FM USVs, are elicited by activation of sites all along the mesolimbic dopaminergic pathway, including the VTA, anteromedial hypothalamus, preoptic area, and the NAc [11,12]. Although the frequency ranges and spectral dynamics of the emitted USVs differ, similar mechanisms also exist in mice [13,14]. Animal models using both mice and rats are utilized in a broad number of research areas, including mental health and addiction [15].

Analysis of rodent USVs is a foundational technique that underlies a diverse cross-section of mental health and addiction research [16,17,18,19,20,21,22,23,24,25,26,27,28]. However, this technique is currently underutilized because USV tabulation is time-intensive, manual, and costly—each two minutes of recorded rodent vocalizations can require up to an hour to hand-score depending upon the number of calls present. Hand-scoring requires extensive prior training before a scorer reaches the necessary level of proficiency, and all too often, this training is sub-optimal, reliability is left unchecked, and large individual differences in hand-scoring results. Notably, even with proficient hand-scorers, the intensive manual nature of the analysis limits researchers’ ability to employ the effective and nuanced experimental designs (e.g., multi-hour and/or multi-session experiments) needed to fully explore mental health and addiction related topics. In addition, with the increased predictive success of acoustic parameters of USVs over discrete call classification, tools are needed to automate this process [15,29,30,31,32]. This represents a bottleneck for current USV researchers and a barrier to entry for other researchers as well.

Recognizing that automation can alleviate the USV analysis bottleneck, several research groups have developed automated USV detection and classification algorithms, including WAAVES [32], XBAT [33], DeepSqueak [34], and MUPET [35]. WAAVES has enabled Duvauchelle and colleagues to rapidly examine emotional profiles in rats across a broad range of experimental settings in a fraction of the time required with manual analyses [30,32,36,37,38,39]. However, the main weakness of all of these automated USV scoring algorithms is that each is customized to specific environmental recording conditions and types of animals (e.g., rats vs. mice) and requires end user trial-and-error-driven thresholding and/or extensive training data to ensure accurate results. These algorithms cannot be broadly applied to data collected in other sound environments without tailoring, thus, impeding research progress.

In this manuscript, we present a snapshot of the development and testing of a new web-based automated USV scoring tool called Acoustilytix (see http://acoustilytix.com/; 30 May 2021). Acoustilytix is environment-agnostic and implements machine learning methodology in the USV detection and classification process (We are in the process of applying this approach in mice). The ongoing development of Acoustilytix is funded through the National Institutes of Mental Health Small Business Technology Transfer (STTR) program, whose mission is to support commercialization of products developed through a partnership between research institutions and industry (It is for this reason that many algorithmic details cannot be presented). The ongoing development of Acoustilytix derives from a unique collaboration between the Cornerstone Research Group (CRG), a high-technology research and development company near Dayton Ohio, and Dr. Christine Duvauchelle, an expert in USV animal research in the College of Pharmacy at the University of Texas (UT). The CRG and UT teams were complemented with an international consortium of USV researchers who generously provided their expertise and input, as well as their USV data to be used during software development. The consortium included a dozen USV researchers from around the world who were interested in helping us develop the most effective automated USV scoring solution.

The remainder of the manuscript is organized into two major sections.

The first focuses on the current state of development of the Acoustilytix platform and the rigorous quantitative testing and evaluation of the platform. The current version of Acoustilytix is quite effective at automated call detection, being environment-agnostic and offering a quantitative measure of call detection uncertainty (see Section 3 below). Acoustilytix is less effective when it comes to automated call classification with this still being a work in progress. In this first section, we briefly summarize some of the user features suggested by our consortium members that have been implemented into Acoustilytix, such as rapid automated USV detection, call parsing, detection confidence, call classification, and parameterization from rats in any USV recording environment. For ease of exposition, the main body of this section will offer a high-level summary, with many of the algorithmic and architectural details in Appendix A, Appendix B and Appendix C. Next a detailed analysis of the platform output and a comparison of its effectiveness at automated call detection with the popular DeepSqueak algorithm is presented. Briefly, with no user intervention or tuning, Acoustilytix achieves 93% sensitivity (a measure of how accurately Acoustilytix detects true calls) and 73% precision (a measure of how accurately Acoustilytix avoids false positives) in call detection across four unique recording environments and was superior to the popular DeepSqueak algorithm (sensitivity = 88%; precision = 41%). Acoustilytix is also much less prone to detecting false positives than DeepSqueak. We conclude with a discussion of automated call classification and our progress to date. To anticipate, automated call classification has been challenging. We had to balance the desire to classify multiple distinct call types with the need for large numbers of hand-scored calls for model testing. To date, we have developed a machine learning model that automates the classification of a five-call scheme suggested by one of our consortium members. Call classification accuracy is in the 71–79% range (well above chance) and will continue to improve as more USV files are scored, which will provide more training data for improving model fit. Perhaps more importantly, discussions with our consortium members suggest that they are less interested in a product that is well calibrated to a single call classification scheme but rather would like a product that allows them to upload a large number of hand-scored calls from a classification scheme of their choice that will then be used to train a machine learning algorithm for subsequent automated scoring. In the General Discussion section, we briefly summarize what a product offering like this might look like.

The second major section focuses on a recently developed feature of Acoustilytix that offers a fast and effective automated method for training hand-scorers without the need for an expert hand-scorer to be present. Building upon the fact that some level of hand-scoring competency is needed in any USV lab, even with Acoustilytix, we describe a new feature that leverages learning science principles to optimize hand-scoring training [40,41]. In short, hand-scorers in training can select the “training” feature in Acoustilytix. They are presented with a list of training files that contain calls that have been hand-scored by an expert in the laboratory whose classification serves as the teaching signal. The trainee can select a file and begin by selecting prototypical calls from a menu that they can visualize using a spectrogram or listen to at a reduced rate. Once the trainee is ready to begin the formal call-by-call training process, they are presented, one by one, with a large number of calls (e.g., 1000), which they may analyze visually using a spectrogram or audibly by listening to the audio clip at a reduced rate. For each call, the trainee selects a call classification and is given immediate corrective feedback (based on the expert scorer’s classification). If in error, the trainee is allowed to examine any prototypical calls again and/or make another classification judgement. This process continues until the trainee provides the correct classification for each call and until all calls in the training set have been correctly classified. Immediate corrective feedback of this sort is known to speed learning and enhance proficiency [40,41]. This increases the likelihood that all hand-scorers in the laboratory mimic the scoring behavior of the expert, thus increasing consistency and reliability of USV hand-scoring. We present the results from a reliability study that verifies the effectiveness of the Acoustilytix hand-scorer training approach.

Finally, we conclude the manuscript with a discussion of future Acoustilytix software development plans and how Acoustilytix might be incorporated into and affect the USV research landscape.

## 2. Acoustilytix Program Structure

In this section, we provide a high-level description of the Acoustilytix program structure. Acoustilytix is a web-based platform that allows USV researchers to easily upload wav files; to trigger automated USV call detection and classification; and to output call classifications, counts, and a number of acoustic characteristics of each call to a csv file for the users’ subsequent analysis.

### 2.1. Acoustilytix Web-Based User Interface and Features

An overriding aim of the Acoustilytix web-based interface is to offer a user-friendly experience for USV researchers. Based on extensive discussions with USV consortium members, the website includes separate pages or drop-down menu functions for wav file uploading, sorting, and tracking of wav files; selecting a USV classification scheme (e.g., the 14 call types identified in Wright, et al., 2010) [42]; initiating the automatic detection; manual verification of automated call detection and classification; and download of tabulated results to a csv file. We briefly summarize each of these functions below.

#### 2.1.1. User Interface

Once a research group gains access to Acoustilytix, the principal investigator (PI) in a laboratory creates a username and password, is assigned their own research group, and receives “administrative” privileges. Once logged in to the Acoustilytix platform, a user can select the “upload file” feature and can either drag and drop or use an upload button to select a wav file for upload to the research group. All research group members can access and score files that have been uploaded by any member of the group. Files can be sorted in any number of ways, for example, alphabetically by file name, by file uploader, or by file scorer. In the current version of the platform, users can select one of the default USV classification schemes from a drop-down menu. (As detailed in the General Discussion section, we are currently building the capability of allowing a researcher to create their own call classification scheme. A large number of hand-scored calls from this scheme will be uploaded to Acoustilytix and will be used to train an automated call classification model.) Once a call classification scheme is selected, it will be applied for all subsequent files until a new call classification scheme is selected. A manual verification process has been implemented that allows researchers to review the automated call classification for any calls they choose. Once a wav file is scored by Acoustilytix, it is available for download as a csv. The file will include the start and stop time, user selected call type classification, and a large number of acoustic parameters associated with each call.

#### 2.1.2. Acoustilytix Features

The two primary features of Acoustilytix are automated call detection and call classification. Because different laboratories or individuals within a laboratory might implement a different call classification scheme, users can select unique call classification schemas to be used during the automated call classification and/or manual verification process.

Acoustilytix automatically begins parsing the audio file and detecting calls upon file upload. Automatic call detection requires three distinct processes—initial detection and file parsing, call isolation and detection confidence, and call parameterization.

*Initial Detection and File Parsing*: Two of the primary guiding tenets of the Acoustilytix automated detection algorithm are: (1) most of the recording time and frequency bins can be attributed to background noise and (2) USVs are connected in time, frequency, acoustic power, or some combination of these. Thus, Acoustilytix begins by estimating an overall measure of the background noise and identifies likely calls as those segments in the file with connected time, frequency, acoustic power, or some combination that is distinct from background noise. Possible calls are expanded by several milliseconds to capture both ends of any potential calls. These expanded segments are organized into a list where any overlapping segments are coalesced together. These segments with potential calls are then passed into the call isolation module. A detailed explanation of this process is included in Appendix A.

*Call Isolation and Detection Confidence*: To isolate calls and differentiate them from background and noise, Acoustilytix uses a combinatorial approach applying several filters and algorithms. These are combined in various ways to differentiate the call from the background and to assign a detection confidence value (percent certainty). The details can be found in Appendix B.

*Parameterization*: Once the calls have been isolated, descriptive parameters can be calculated; the current version of Acoustilytix calculates over 200 parameters for each call. These include parameters such as maximum, minimum, and median frequency; maximum acoustic power (and frequency at which this occurs); and median acoustic power along the call maximal ridge. A review of commonly referenced call types for both rat and mice reveals that temporal variations, i.e., slopes, jumps, etc., appear indicative of many call types [14,42]. Using the ridges from the isolation step as an initial guide, many temporal-varying parameters are also calculated, including max, min, median, and standard deviation of slopes for not only the whole call but also the beginning, middle, and end of the call. Jumps and gaps are also tabulated, as well as a few measures of how quickly slopes change over time (slope variability), which relates to “trill” type calls. The current iteration of the software outputs a sub-set of these parameters in the csv file for researchers. We fully expect the use of these parameters in USV research will become more common [29,30,43].

Manual verification of automated call detection is not a mandatory step in the Acoustilytix process. Even so, and especially since software development is ongoing, we urge researchers to incorporate manual verification into their workflow. During software development, the CRG and UT teams worked iteratively to ensure that the manual verification process was easy to implement and streamlined. The result of this development effort is that the platform currently offers three data formats for exploring and interacting with the data in each file-card, tile, and table. Briefly, the “card” format presents a single segment/call combination with the ability for users to play back the audio segment while a line moves through a responsive spectrogram. From this screen, users can modify the call bounds, mark an auto-detected call as a false positive, or provide a USV classification type. The “tile” format provides an infinite scrolling list of the segment and call combinations for a broader overview. The call parameters and any user-selected call classification are shown on the tile. Tiles can be filtered to include all calls or only calls above a threshold detection confidence (i.e., percent certainty) for detection. Finally, the “table” format provides parameterization details for each of the calls, as well as any user added comments or classifications. These data are able to be downloaded as a csv file for each scoring session. Scoring data are organized in “scoring sessions” with data linked to the user, wav file, and call classification scheme. Progress for each scoring session is tracked, and users can easily resume scoring where they left off. In addition, each file can be scored separately by multiple users or using multiple classification schemes. Multiple users can score the same file, and the results can be easily compared to assess inter-rater reliability.

## 3. Acoustilytix Validation Results

### 3.1. Acoustilytix: Detection Statistics

To generate detection statistics, the output from Acoustilytix was compared to compiled data from hand-scored USV files and to the output from DeepSqueak. The time stamps were matched between datasets, and then, calls from the Acoustilytix or DeepSqueak dataset were given one of the following three designations:

- Detected: These are defined as calls detected by hand-scorers and by Acoustilytix (or DeepSqueak).
- Missed: These are defined as calls detected by hand-scorers but not detected by Acoustilytix (or DeepSqueak).
- False Positive: These are defined as calls not detected by hand-scorers that were detected by Acoustilytix (or DeepSqueak).

Two metrics were used to quantify the success of Acoustilytix (or DeepSqueak) at matching the hand-scored data.

- Sensitivity = Detected/(Detected + Missed): This is a measure of how many of the hand-scored calls are correctly detected by Acoustilytix (or DeepSqueak).
- Precision = Detected/(Detected + False Positive): This is a measure of how many hand-scored calls Acoustilytix (or DeepSqueak) detects but also the number detected that are not detected by the hand-scorers.

We examined the sensitivity and precision from different experimental conditions across different recording environments. These results are summarized in Table 1.

For Acoustilytix, the sensitivity (accurate detection of calls compared to hand-scored) was above 90% in all cases, with an average of 92.7%. This means that Acoustilytix correctly detected over 90% of the calls noted via hand-scoring in a variety of experimental and recording conditions without any user intervention or tuning. For DeepSqueak, the sensitivity was 87.7% on average for 18,770 USV calls. Compared with Acoustilytix, DeepSqueak’s sensitivity had a *p*-value less than 0.00001, which showed a significant difference.

Precision is a critical metric for automated detection success, as the number of false positive USVs should be minimized. As the results in Table 1 suggest, precision was lower than sensitivity in all cases, ranging from 59.5–83.6% with a weighted average of 73.2%. DeepSqueak had an average precision of 41.0% with a *p*-value less than 0.00001 when compared with Acoustilytix’s 73.2% average precision.

In developing and evaluating the USV detection algorithm, we realized that some metric of call detection certainty (or confidence) could be of value to USV researchers. We developed such an algorithm, and these values are part of the csv output file. The details of the algorithm are being evaluated as potential intellectual property (IP) and, thus, cannot be presented in detail here. At a high level, call detection certainty is a function of acoustic power of the parsed signal relative to the background power, with greater acoustic power for the parsed signal relative to the background being associated with greater call detection certainty.

In the next iteration of Acoustilytix, we plan to incorporate a new feature into the detection workflow that will allow the USV researcher to set a threshold on the call detection certainty so that all calls below the research-selected threshold will be automatically made available for manual verification.

### 3.2. Acoustilytix: Call Classification Statistics

The goal of this section is to report the progress toward developing an automated USV call classification machine learning algorithm. Our original goal was to develop an automated USV call classification machine learning algorithm that could accurately classify 20 or more unique call types that are used in the literature. These might include the 15 call types identified by Wright et al. [42] but also more complex waveforms, including dysphonation and frequency shifts, which may have particular relevance to translational research [44,45]. After some initial attempts, this was not deemed feasible in a reasonable amount of time. In addition, and based on discussions with our USV consortium members, we were informed that researchers were more interested in having the ability to upload their own hand-scored USV files that could be used to train an automated scoring algorithm that they could use exclusively.

**Figure 1:**
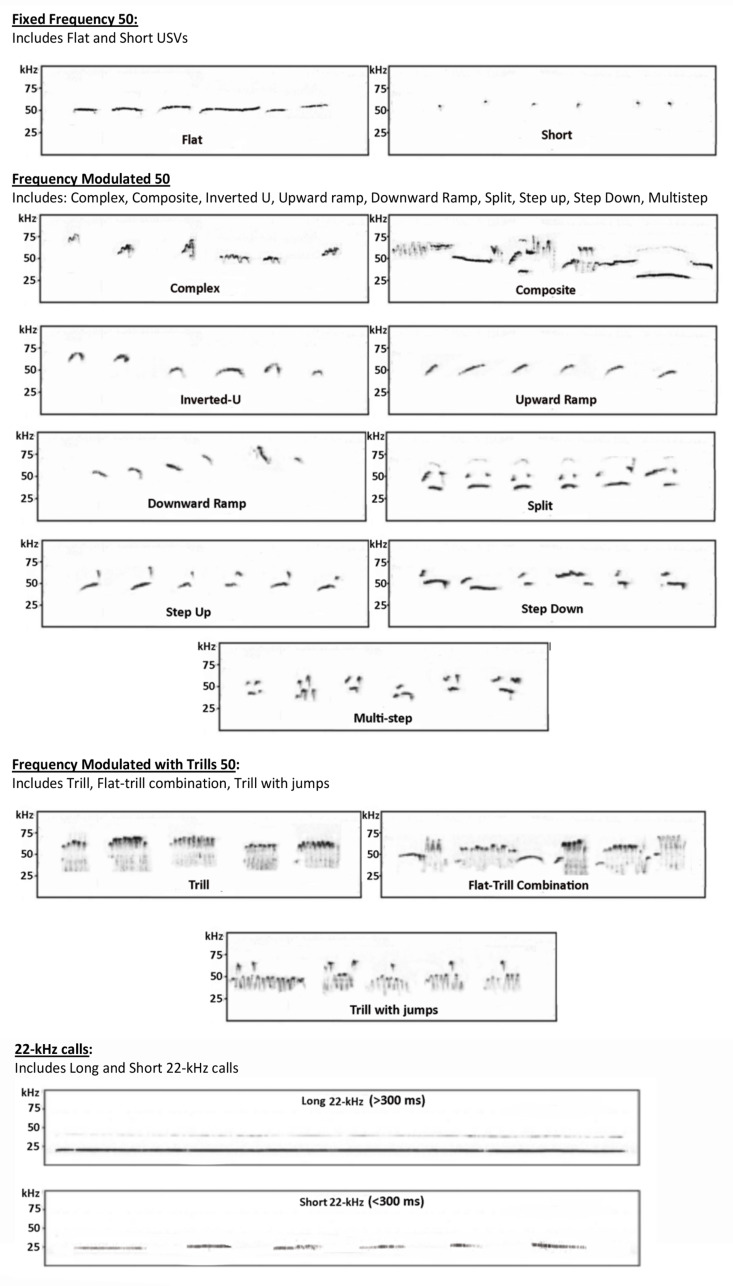
Representative spectrograms for each of the call types in Table 2.

The first step in achieving this aim was to identify which of many machine learning algorithms to implement. Although a number of machine learning algorithms were explored, we settled upon a random forest algorithm whose parameters were estimated from hand-scored USV call classification data. For the initial validation test, we wanted to balance the need to classify a broad range of calls with the need to have the statistical power to train and test the model. We settled on a five-call classification scheme that has been used by one of our consortium members and represents a composite of an existing call classification scheme by Wright et al. [42]. The mapping from the Wright categories to the five-call composite is presented in Table 2. Representative call spectrograms are displayed in Figure 1. It is important to note that we are not arguing that this is the best call classification scheme to be used in translational research. Rather, the goal is to verify that a random forest algorithm can be used to build an automated hand-scoring tool with reasonable accuracy.

#### Results

A machine learning algorithm was trained using a supervised random forest classification model where Dr. Duvauchelle’s most experienced rater’s call classifications were used as the supervised teaching signal for the model. The dataset consisted of 931 rat calls. Table 3 shows the number of each call type in the dataset.

The experimental dataset was split into a training dataset consisting of 70% of the calls and a test dataset with the remaining 30% to validate the model’s performance and to prevent overfitting. The model features consisted of the parameters that were calculated by the call detection algorithm discussed in the Parameterization section of this paper. Feature selection methods were employed to down-select the number of predictors in the model and to prevent overfitting. A grid-based approach was used to tune the random forest model parameters and to identify the model that yielded the highest test data accuracy.

We began the evaluation process by tallying the number of calls of each type that fall in each of the following four categories:

- True Positive (TP): The hand-scorer selected call type “A”, and the classifier selected call type “A”. The hand-scorer and classifier agreed that call type “A” was present.
- True Negative (TN): The hand-scorer did not select call type “A”, and the classifier also did not select call type “A.” The hand-scorer and classifier agreed that call type “A” was absent.
- False Positive (FP): The hand-scorer did not select call type “A”, but the classifier selected call type “A.” The hand-scorer and classifier disagreed and the hand-scorer’s call type selection was considered the true call type.
- False Negative (FN): The hand-scorer selected call type “A”, but the classifier did not select call type “A.” The hand-scorer and classifier disagreed and the hand-scorer’s call type selection was considered the true call type.

These were then used to compute the five model performance metrics displayed in Table 4: These include precision, recall, F1-score, support, and accuracy, which are calculated from the TP, TN, FP, and FN values as follows. The F1-Score is the harmonic mean of precision and recall and gives a better measure of the incorrectly classified cases than the Accuracy metric.

- Precision = TP/(TP + FP)
- Recall = TP/(TP + FN)
- F1-score = 2 * (Precision * Recall)/(Precision + Recall)
- Support = count
- Accuracy = (TP + TN)/Total Population

**Table 4:** Evaluation of the five-call composite classification model performance on the test dataset.

Five-Call Type	Precision	Recall	F1-Score	Support
Fixed Frequency 50	0.77	0.69	0.73	49
Frequency Modulated 50	0.73	0.87	0.80	12
Frequency Modulated with Trill 50	0.81	0.62	0.70	42
Long 22-kHz	0.92	0.87	0.89	53
Short 22-kHz	0.82	0.75	0.78	24
**Overall Accuracy/Support (N-size)**			**0.79**	**280**

Precision, recall, F1-score, and accuracy fall between 0 and 1 where values close to 1 indicate strong model performance. Table 4 shows good performance on all of the five call types across all performance metrics with an average accuracy of 79%, which is well above chance level (20%).

For comparison, Table 5 shows how well the model performed on the training data.

Again, model performance was quite good on all performance metrics with an accuracy for the training dataset of 87%. We used a kappa statistic to compare the model to the expert hand-scorer’s full data set. Kappa statistics range from 0 to 1 with higher values denoting greater agreement. The model yielded a kappa = 0.79, suggesting strong agreement between the model and the expert hand-scorer. As new data are scored, the model fit will continue to improve and further optimize (We also fit the model with over 30,000 hand-scored calls from a team of six hand-scorers. Seventy percent of the data were used to train the model and 30% to test the model. Test data accuracy was 71% as compared to the 79% with the single expert hand-scorer, and training accuracy was 88% as compared to 87% with the single expert hand-scorer.).

### 3.3. Acoustilytix Validation Results General Summary and Future Directions

In this section we evaluated the effectiveness of Acoustilytix as an automated USV call detection tool and compared it with the currently popular DeepSqueak algorithm. We also examined the ability of a random forest machine learning algorithm to serve as the foundation for automated call classification. We gained a number of important insights from this evaluation.

First, our automated USV detection algorithm was not significantly impacted by background noise. Background noise can obfuscate USVs from both hand-scorers and existing automated call classification solutions. Acoustilytix’s proprietary detection algorithm maintained excellent performance even in the presence of background noise. In fact, we showed that USVs missed by hand-scorers during initial scoring were subsequently identified by Acoustilytix and confirmed as true calls by hand-scorers. With no user intervention or tuning, Acoustilytix achieved 93% sensitivity (a measure of how accurately Acoustilytix detects true calls) and 73% precision (a measure of how accurately Acoustilytix avoids false positives) in call detection accuracy across four unique recording environments. Critically, Acoustilytix also generated a call detection certainty metric that reflected the confidence in the model call detection decision. In future iterations of Acoustilytix, we plan to incorporate this into the optional manual call verification process so that USV researchers have the option to manually verify any calls with a detection certainty below some threshold selected by the USV researcher.

Second, Acoustilytix had significantly better sensitivity and precision than DeepSqueak (sensitivity = 88%; precision = 41%) across the four studies analyzed, which included four different environments. DeepSqueak had a high false positive rate across the four studies, which posed a challenge to hand-scoring.

Third, our automated USV call classification algorithm performed quite well on the five-call composite outlined in Table 2. While there is room for continuous improvement, and we have a plan for implementing this (see future directions), Acoustilytix accurately classified 79% of the hand-scored data from nearly 1000 calls classified by an expert into the five-call types. This is well above chance (20%). As additional data are uploaded to the Acoustilytix platform, and as hand-scorers modify or endorse the Acoustilytix classification, model parameters will continue to be optimized in the interest of increased accuracy.

Fourth, and based on conversations with our USV consortium members, it is clear that they prefer an automated call classification platform that allows them to upload a large number of hand-scored USV files that could be used to train an automated scoring algorithm that they could use exclusively. Our progress with the random forest algorithm displayed in Table 4 and Table 5 suggest that this feature can be effectively implemented in the next iteration of Acoustilytix.

Fifth, and perhaps most exciting, is the fact that over 200 acoustic parameters associated with each detected USV are being computed and can be outputted in the csv files. As many research groups have shown, including our own [14,30,46,47,48,49,50,51], the USV acoustic parameter values are highly predictive of a number of addiction and other health related outcomes. It is very likely that the ease of acquisition of these parameters when using Acoustilytix will quicken the pace of research focused on acoustic parameters.

## 4. Acoustilytix Hand-Scoring Training Feature

As outlined above, the need for highly proficient, fast, and consistent hand-scoring is a clear bottleneck for the use of USVs in research. Few USV researchers are satisfied with their current hand-scoring training procedures, and most report their desire for an automated approach to hand-scorer training.

To accurately hand-score a USV, the scorer must detect the call as distinct from noise in a spectrogram, then, must visually inspect the USV and listen to it to accurately classify the call. This mental representation must then be compared with either visual and auditory mental representations of prototypical calls of each type or actual visual and auditory spectrograms of prototypical calls from each type. The scorer must then assign the call to a USV category.

The best way to train this type of complex visual/auditory matching task is to have a trainee inspect a potential call, classify it, then receive immediate feedback from an expert hand-scorer on the correctness of their classification. This process should be repeated several hundred times to build expertise. Unfortunately, this is time-consuming and costly and, thus, rarely occurs. More commonly, a hand-scorer in training is shown examples along with verbal descriptions of each call type. They are then asked to score a large number of calls (e.g., several hundred), and they only receive corrective feedback after the fact. This is suboptimal, as the best time to strengthen or weaken the neural connection between the stimulus (call to classify) and response (classification) is to provide feedback immediately following the classification [40,41,52].

We recently developed a new function in Acoustilytix to automate the hand-scoring training process that builds upon what is known about learning to optimize the speed, accuracy, and consistency of the resulting hand-scoring capability. This function includes an initial USV call familiarization phase in which a hand-scorer in training can view and listen to spectrograms of numerous calls from any selected call category. Learners can do this at their own pace for as long as they want. This familiarization tool remains available to hand-scorers at all points in time. Once a hand-scorer is ready to practice hand-scoring, they retrieve a USV file from the Acoustilytix platform that has been “tagged” as a training file. This training file has been hand-scored by an expert from the USV laboratory. The expert simply generates a csv file that includes the correct response, uploads it to the platform, and tags it as a training file. Once the hand-scorer in training begins, the first call is presented. The hand-scorer can look at the call and use all of the tools outlined above (e.g., zoom), as well as listen to the call. “Flashcards” of prototypical calls are available for the hand-scorer to view. Once they are ready to classify, they select a call category from a drop-down menu, and they receive immediate feedback from the platform. If their classification is correct, they go on to the next call. If their classification is incorrect, they can either select a new call category, or they can view the prototypical call flashcards and can compare them with the to-be-classified call until they are ready to generate their own classification. This process continues until they classify all calls in the training file. Once complete, an accuracy rate (based only on the first call classification) is displayed. Multiple training files can be scored until the hand-scorer is proficient.

### Acoustilytix: Hand-Scoring Trainer Inter-Rater Reliability

A group of five individuals were given traditional (unstructured training), with each subsequently classifying several thousand calls into the five call types described above over the course of several months (ranging from 6000–31,000 calls classified by each hand-scorer). At a later date, all of these individuals were asked to individually score a single test file with approximately 1000 calls that had been hand-scored by an expert (test following unstructured training). They were given one week to complete this task.

We used a kappa statistic to compare each hand-scorer with the expert hand-scorer. An example of the kappa statistic calculation can be found in Appendix C. The kappa statistics for the initial group of five individuals in the test following unstructured training is presented in the top portion of Table 6. Notice that the kappa values are moderate following unstructured training ranging from 0.30–0.55 with a mean of 0.42.

Next, we asked two individuals with no prior USV hand-scoring experience to complete Acoustilytix training with approximately 1000 calls, and they were given the same Acoustilytix test. The kappa statistics for the two Acoustilytix-only trained individuals are presented at the bottom of Table 6 (Hand-scorer 6–7). The kappa values were 0.42 and 0.47 with an average of 0.445. This is comparable to the average kappa value for the five extensively trained hand-scorers and, in fact, is slightly higher (0.445 vs. 0.42).

Given the success of Acoustilytix training with the two novice hand-scorers, we asked the five experienced hand-scorers to complete Acoustilytix training with approximately 2000 calls, and they were given the same Acoustilytix test. The kappa statistics for the five Acoustilytix-only trained individuals are presented in the right-most column of Table 6. The kappa values ranged from 0.30–0.75 with an average kappa value of 0.60. Four out of five experienced hand-scorers had significantly better kappa statistics after the Acoustilytix training.

These findings suggest that hand-scoring training in which the learner is given immediate call-by-call feedback, and where the teaching signal is based on the call classification from an expert hand-scorer, leads to more accurate and consistent hand-scoring. Across all seven hand-scorers, and following Acoustilytix training, the average kappa value was 0.55. Further training would likely increase this value. USV researchers could set a minimum threshold on this value if they like and deem hand-scorers “qualified” only when they exceed that threshold. Refresher training could also be incorporated to ensure consistency and accuracy of hand-scoring.

## 5. General Discussion and Future Directions

Ultrasonic vocalizations in rodents provide a window into emotional and neural processing. These processes are directly involved in many mental health disorders and addictions. Despite the potential of USV analysis in mental health and addiction research, one huge bottleneck exists—namely, the time-intensive, manual and costly nature of USV hand-scoring—specifically, call detection and call classification.

In this report, we provide a snapshot of our current development and testing of Acoustilytix, a web-based automated USV scoring tool that is currently environment-agnostic and implements machine learning methodology in the USV detection and classification process.

Based on discussions with our USV consortium, we identified and implemented a number of features to make the platform easy to use. These include the ability to easily upload USV files, output csv files, and the ability to manually verify or modify an automatically detected call. The initial test of the call detection algorithm in Acoustilytix was promising. With no user intervention or tuning, Acoustilytix achieved 93% sensitivity (a measure of how accurately Acoustilytix detects true calls) and 73% precision (a measure of how accurately Acoustilytix avoids false positives) in call detection accuracy across four unique recording environments and was superior to the popular DeepSqueak algorithm (sensitivity = 88%; precision = 41%). Long calls and short calls are scored separately in DeepSqueak because the functions for each algorithm work differently. While analyzing and comparing the long and short call files, we noticed that DeepSqueak can detect long and short calls that overlap either partially or fully in time. This can create confusion regarding the spectral dynamics of each USV and make analysis more challenging. Because Acoustilytix’s call detection algorithm does not require separate analyses of the USV recording files, this problem is avoided. The ability to accurately detect USVs without any user intervention or tuning addresses a major barrier to broad adoption of USV models in addiction and mental health research. Once we incorporate the quantitative metric of call detection certainty into the user facing Acoustilytix dashboard, researchers will be able to set a threshold for manual verification that is fast, accurate, and flexible for users.

We also examined USV call classification accuracy in a five-call composite classification scheme derived from the Wright et al. [42] call categories. Automated call classification accuracy using a random forest machine learning algorithm was 79% for the five-call composite. These accuracy rates are well above chance, but as additional data are uploaded to the Acoustilytix platform, and as hand-scorers modify or endorse the Acoustilytix classification, model parameters will continue to be optimized in the interest of increased USV call classification accuracy. The validation of the random forest model sets the stage for the next iteration of the automated USV call classification algorithm. As requested by our USV consortium members, we plan to incorporate a new feature that allows researchers to upload a large number of hand-scored USV files that could be used to train an automated scoring algorithm that they could use exclusively.

Perhaps most exciting is the fact that over 200 acoustic parameters associated with each detected USV are being computed and can be outputted in the csv files. As our research group and others have shown [31,37,42,53,54,55,56,57,58,59], the USV acoustic parameters are highly predictive of a number of addiction and other health related outcomes. We expect that the ease of acquisition of these parameters when using Acoustilytix will quicken the pace of research focused on acoustic parameters.

We also introduced a recently developed feature of Acoustilytix that offers a fast and effective way to train hand-scorers and is built upon a foundation of learning science [40,41]. Critically, trainees are presented with several hundred USVs one at a time, are asked to classify the call, and receive immediate corrective feedback based on the hand-scoring from an expert in the lab. We tested this approach in seven novice hand-scorers and showed that their post training call classifications were highly correlated with the expert (inter-rater reliability: kappa statistic ranged from 0.30–0.75, average = 0.55). This approach increases the likelihood that all hand-scorers in the laboratory mimic the scoring behavior of the expert, thus, increasing consistency and reliability of hand-scoring.

We now briefly discuss some future directions and further refinements planned for Acoustilytix.

*Call Detection*: With no user intervention or tuning, we were able to achieve high levels of call detection accuracy that were superior to the popular DeepSqueak algorithm. Even so, we are exploring the application of machine learning models in the USV call detection process to further eliminate false positives. As more USV wav files from other research groups are uploaded to the platform and are run through the detection algorithm, this model can be further tuned to provide better separation of valid calls from false positives.

*Call Classification*: Although automated call classification for the five-call type classification scheme was well above chance, there is still room for improvement. Having explored numerous machine learning types, we are confident that the supervised random forest approach is a good approach, but we need a significantly larger pool of data with hand-scored calls from experts to further improve the predictions from the algorithm. This process is ongoing. As we outline in this manuscript, we began with an expert hand-scorer scoring 1000 calls or so. We used that to train the model. Every time the expert scored a new file, that triggered a model refit so that the fit improved based on the larger set of data. Users can use the model developed from our expert, or they can use their own expert to train the model on a specific call classification scheme. Ultimately, we will use all of these data to train the model using expert scorers from multiple research groups using the same classification scheme. This is beneficial to the whole USV community because it will lead to more uniformity and consistence in scoring within and across research groups, making the research findings more reliable and reproducible.

*Acoustic Parameters*: Several research groups [10,14,42,46,47,48,49,50,51,53,54,56,57,59,60], including our own [29,30,31,32,36,37,38,39,43,61,62,63,64], have shown that acoustic parameters are predictive of addiction and mental health related outcomes. For example, our own research group has shown that acoustic parameter values are predictive of whether a call is emitted by a high vs. low alcohol drinking rat or whether that rat is male or female [37,43], as well as a number of other interesting distinctions. Acoustilytix currently computes over 200 acoustic parameters for each call that can be analyzed by USV researchers. Future work should consider applying multi-dimensional similarity-based approaches to create novel call classification schemes that are strictly data driven, in addition to exploring the predictive nature of specific or groups of parameters.

## 6. Conclusions

This report offers a snapshot of the development of a web-based automated USV detection and classification software solution called Acoustilytix. Acoustilytix accelerates throughput of USV scoring by automating the call detection and classification process. Acoustilytix achieves high detection accuracy across four distinct environments without manual tuning or calibration and is superior to the currently popular DeepSqueak algorithm in sensitivity for three out of four environments and superior in precision in all four environments. Automated call classification was adequate yielding 79% accuracy, but more work is needed. A feature that allows researchers to upload a large number of hand-scored USV files that could be used to train an automated scoring algorithm that they could use exclusively is in demand and under development. The development of an automated hand-scoring feature is innovative and has been well received by the USV community. Taken together, Acoustilytix has the potential to speed the process of USV detection and classification in the interest of developing and testing animal models of mental health and addiction.

## Figures and Tables

**Table 1:** Sensitivity and precision of Acoustilytix and DeepSqueak for four experimental conditions across four recording environments.

			Sensitivity			Precision		
Recording Environment	Brief Description of Study Manipulation	# of Hand-Score Detected USVs	Acoustilytix	DeepSqueak	*p*-Value	Acoustilytix	DeepSqueak	*p*-Value
1	Cocaine	8552	92.5	91.5	0.016	74.5	42.0	<0.00001
2	Ethanol	1720	94.0	92.1	0.029	72.0	65.8	0.00008
3	Morphine	5771	90.6	75.8	<0.00001	83.6	33.1	<0.00001
4	Sex	3462	96.4	97.5	0.0078	59.5	46.1	<0.00001

**Table 2:** Call types in the Wright et al. [42] classification scheme and their mapping to the five-call composite with representative spectrograms from Acoustilytix.

Wright et al. [42]	Five-Call Composite
Flat	Fixed Frequency 50
Short	
Upward Ramp	Frequency Modulated 50
Downward Ramp	
Split	
Step Up	
Step Down	
Multi-step	
Inverted-U	
Complex	
Composite	
Trill	Frequency Modulated with Trills 50
Flat-Trill Combo	
Trill with jumps	
22-kHz call	Long 22-kHz
	Short 22-kHz (>300 ms)

**Table 3:** Number of each of five call types in the five-call composite dataset classified by an expert scorer.

Call Types	Count
Fixed Frequency 50	202
Frequency Modulated 50	342
Frequency Modulated with Trill 50	132
Long 22-kHz	184
Short 22-kHz	71

**Table 5:** Evaluation of the five-call composite classification model performance on the training dataset.

Five-Call Type	Precision	Recall	F1-Score	Support
Fixed Frequency 50	0.89	0.80	0.84	153
Frequency Modulated 50	0.82	0.93	0.87	230
Frequency Modulated with Trill 50	0.92	0.81	0.86	90
Long 22-kHz	0.92	0.92	0.92	131
Short 22-kHz	0.86	0.77	0.81	47
**Overall Accuracy/Support (N-size)**			**0.87**	**651**

**Table 6:** Kappa statistics for experienced and novice hand-scorers relative to an expert scorer.

Hand-scorer	Test Following Unstructured Training	Test Following Acoustilytix Training	*p*-Value
Experienced Scorers			
1	0.42	0.29	<0.00001
2	0.55	0.60	0.031
3	0.30	0.75	<0.00001
4	0.36	0.69	<0.00001
5	0.49	0.64	<0.00001
Novice Scorers			
6	NA	0.47	NA
7	NA	0.42	NA

## Data Availability

Not applicable.

## Appendix A. Details of Initial Detection and File Parsing

To determine where a call is most likely to exist, a coarse spectrogram (STFT) of the entire file is calculated. This coarse spectrogram is processed to account for differences due to the recording setup, which can result in varying acoustic power with respect to frequency bins. A maximum acoustic power is then derived from this processed spectrogram at each time interval, resulting in a one-dimensional (1D) signal. To determine where possible calls are located, calls are treated as outliers in this 1D signal.

## Appendix B. Call Isolation and Detection Confidence

To isolate calls and differentiate them from background and noise, Acoustilytix uses a combinatorial approach, applying several filters and algorithms. The details of the algorithm are being evaluated as potential IP and, thus, cannot be presented in detail here. These filters are combined in various ways to extract the calls out of the background and provide a detection confidence value (percent certainty)

## Appendix C. Example Kappa Statistic Calculation

In calculating the kappa statistic between two scorers, USVs that Acoustilytix missed were not included in the calculation. We were unable to include user-added calls in the analysis because the expert scorer did not add calls when analyzing calls in some recording files. Acoustilytix, at times, detects false USVs. Both scorers can assign the “false positive” category to these USVs. The false positive category was included in the kappa statistic calculation. The method used to calculate the kappa statistic can be found in the University of Nebraska-Lincoln Bivariate Statistics Hand-Computation Cache (2021, February). To begin calculating the kappa statistic, a confusion matrix is generated. The confusion matrix is a 5 × 5 matrix where each row represents one of the five call types, and each column represents the same five call types. The rows denote the classification for the individual hand-scorer and the columns for the expert hand-scorer. For each call classified, the value in one cell of the 5 × 5 confusion matrix is incremented. For example, if both the individual and expert classify a call as a Short 22-kHz then the cell in the confusion matrix that represents Short 22-kHz for the individual hand-scorer and Short 22-kHz for the expert is incremented by 1. This process is repeated until all calls are represented in the confusion matrix.

Table A1 shows an example of a confusion matrix for 748 rat calls that were each scored by one individual and by the expert.

**Table A1:** Example of a confusion matrix comparing call types between two raters.

		Rater 1					
		Fixed Frequency 50	Frequency Modulated 50	Frequency Modulated with Trill 50	Long 22	Short 22	Row Totals
Rater 2	Fixed Frequency 50	110	75	3	0	1	189
	Frequency Modulated 50	32	305	16	0	0	353
	Frequency Modulated with Trill 50	6	126	60	0	0	192
	Long 22	2	1	0	1	0	4
	Short 22	0	0	0	0	10	10
	**Column Totals**	**150**	**507**	**79**	**1**	**11**	**748**

Interpreting the confusion matrix is straightforward. On-diagonal elements represent calls where the two raters are in agreement, and off-diagonal elements represent calls where the two raters are not in agreement. For example, Rater 1 and Rater 2 agreed that 110 calls were the “Fixed Frequency 50” call type, but Rater 1 assigned 75 calls to the “Frequency Modulated 50” call type that Rater 2 assigned to “Fixed Frequency 50.”

The kappa statistic calculation uses the row sums, column sums, and overall call count. These are shown in the final column and final row in the example in Table A1. The overall total is the same whether you sum the row totals or column totals and is 748 in this example.

Next, the total number of agreements are found by summing the values in the diagonal cells of the confusion matrix. In this example,

*Σ agreements* = *Σ a* = 110 + 305 + 60 + 1 + 10 = 486

The expected frequency for the number of agreements is now calculated for each agreement along the diagonal by multiplying the row total by the column total and dividing by the overall total (see Table A2). For example, row #1 multiplied by column #1 and divided by the overall total is:

*expected frequency* = *ef* = 189 ∗ 150/748 = 37.90

**Table A2:** Expected frequencies calculated from confusion matrix in Table A1.

	Expected Frequency
Fixed Frequency 50	37.90
Frequency Modulated 50	239.27
Frequency Modulated with Trill 50	20.28
Long 22	0.0053
Short 22	0.1470

The sum of the expected frequencies of agreement by chance is the sum of the expected frequencies. For example:

*Σ expected frequencies* = *Σ ef* = 37.90 + 239.27 + 20.28 + 0.0053 + 0.1470 = 297.60

The kappa statistic can now be calculated with the Formula:

*K* = (*Σ a* − *Σ ef*)/(*N* − *Σ ef*) = (486 − 297.60)/(748 − 297.60) = 0.418
